# Association Between High-Level D-Dimer at Admission and Early Intubation in Patients With Moderate Traumatic Brain Injury

**DOI:** 10.1089/neur.2023.0068

**Published:** 2023-10-25

**Authors:** Qi Zhang, Hong Min Kuang, Du Juan Qiao, Xiang Lin Zhong, Jia Jia Kang, Rui Na Ma, Min Li

**Affiliations:** ^1^ College of Basic Medicine, The Fourth Military Medical University, Xi'an, China.; ^2^ Department of Critical Care Medicine, The Second Affiliated Hospitals, The Fourth Military Medical University, Xi'an, China.; ^3^ Department of Neurosurgery, The Fourth Military Medical University, Xi'an, China.; ^4^ Department of Pulmonary and Critical Care Medicine, The Fourth Military Medical University, Xi'an, China.

**Keywords:** D-dimer, endotracheal intubation, moderate traumatic brain injury, risk factor

## Abstract

It is unclear who can benefit from tracheal intubation in the moderate (mTBI) traumatic brain injury (TBI) population. Given that mTBI patients are conscious, intubation can cause intense stress, possibly triggering neurological deterioration. Therefore, identifying potential risk factors for intubation in mTBI patients can serve as a valuable clinical warning. We sought to investigate whether elevated D-dimer is a possible risk factor for intubation in mTBI patients. Using the STROBE statement, adult patients with isolated TBI (Glasgow Coma Scale [GCS] score 9–13) treated at a high-volume neurotrauma center between January 2015 and December 2020 were reviewed. The demographics, clinical presentation, neuroimaging, and laboratory information were collected based on the patients' electronic medical record. D-dimer values were assessed from serum when patients were admitted to the hospital. The primary study end-point was that the mTBI patient was intubated within 72 h upon admission. A total of 557 patients with mTBI were finally included in this study. Of these, 85 (15.3%) patients were intubated. Multi-variate logistic regression analysis showed that high-level D-dimer (≥17.9mg/L) was significantly associated with early tracheal intubation in mTBI patients (odds ratio, 3.10 [1.16–8.25]; *p* = 0.024) after adjusting for age, sex, GCS scores, Marshall scores, and Injury Severity Scores. Sensitivity analysis showed that high-level D-dimer had a robust correlation with intubation in the different subgroups or after propensity score matching. High-level D-dimer on admission is an independent risk factor for early tracheal intubation in isolated mTBI patients.

## Introduction

Traumatic brain injury (TBI) imposes a significant burden on both society and families in China. It is one of the primary causes of death and disability related to injuries, and it is considered to be responsible for ∼300 hospital admissions and 13 deaths per 100,000 people per year in the country.^1–4^ Among risk factors for TBI patients' death, hypoxia and hypoxemia are independent risk factors that must be prevented.^5^ To prevent hypoxia, patients with a compromised airway and reflexes and a depressed level of consciousness should have their airway secured. This can be done by intubating the trachea to protect the airway and maintain normoxia and normocapnia.^6–8^

Guidelines and clinical protocols for severe TBI recommend comatose TBI patients with Glasgow Coma Scale (GCS) score <8 to be routinely intubated to protect the airway, prevent hypoxia, control ventilation, and improve outcome.^9,10^ However, it is currently unclear whether intubation during moderate TBI (mTBI; GCS 9–13^9,11–13^) provides any benefits. Because mTBI patients are conscious, tracheal intubation can cause intense stress and pain, increase intracranial pressure, and possibly trigger neurological deterioration.^5^ It is difficult to determine the timing of intubation in patients with mTBI. Therefore, identifying independent risk factors for tracheal intubation upon admission in patients with mTBI carries crucial clinical implications and can serve as a valuable warning for clinicians.

D-dimer, a product of fibrin degradation, can be detected in the blood within a few minutes of a TBI.^1^ It serves as a general indicator of coagulation and fibrinolytic systems triggering and can be used as an indirect indicator of thrombus activity.^2^ Elevated D-dimer at admission was not only a strong predictor of deep vein thrombosis and pulmonary embolus, but also an indicator of the need of tracheal intubation and invasive mechanical ventilation in patients with new coronavirus disease 2019 (COVID-19).^3,4^ However, the relationship between an increased level of D-dimer upon admission and the probability of tracheal intubation in patients with mTBI is currently unknown. Here, we sought to investigate whether the D-dimer could serve as a biomarker to help in assessing the need for early intubation in mTBI patients, hypothesizing a positive association between elevated D-dimer and tracheal intubation.

## Methods

### Experimental design, subjects, and location

This was a retrospective, cross-sectional study that reviewed all patients with mTBI who received treatment at Tangdu Hospital, affiliated with the Fourth Military Medical University, between January 1, 2015 and December 31, 2020.

Our retrospective study focused on adult patients with acute isolated mTBI. The inclusion criterion was a clear history of TBI with a GCS score of 9–13^9,11–13^ at admission. Exclusion criteria were: 1) patients younger than 18 years; 2) history of venous or arterial thrombosis or thrombolytic therapy; 3) history of cancer, liver, or renal disease; 4) TBI plus multiple trauma; and 5) gravidity or puerperium.

The study received approval from the Institutional Ethics Board of the Second Affiliated Hospital of the Fourth Military Medical University (K202305-36), and the requirement for informed consent was waived. The methodology followed the recommendations of the Strengthening Observational Studies in Epidemiology Reporting (STROBE) statement.^14^

### Data obtention and main study end-point

Patients' data, including medical records, laboratory results, and radiological images, were collected from the electronic health record (EHR) system of the healthcare facility. D-dimer was measured from the serum of patients' blood samples with Latex Immunoagglutination Assays (SEKISUI Coapresta-2000 Coagulation Analyzer; Sekisui Medical CO., LTD, Tokyo, Japan). The intra-assay coefficient of variation was between ∼3.5% and 4.5%. All the samples were duplicated in two in which one was for testing and the other was kept for 7 days for retesting, if needed.

Three authors carefully reviewed the medical records on separate occasions to collect demographic information, symptoms, and neuroimaging findings from non-enhanced computed tomography scans (such as traumatic hemorrhage, brain contusion, intraventricular hemorrhage, and midline shift [MLS]).

The main study end-point of this research was the tracheal intubation of mTBI patients in the early stay (within 72 h of admission), which was retrospectively collected.

### Exposure and covariates

The primary exposure of interest in this study was the level of D-dimer at admission. Laboratory information was retrospectively collected from the EHR. Other covariates included age, sex, history of smoking or alcohol use, GCS scores, Marshall scores, Injury Severity Scores (ISS), and MLS and comorbidities such as hypertension (HTN), chronic obstructive pulmonary disease (COPD), diabetes mellitus (DM), and coronary heart disease (CAD). Two experienced neurosurgeons independently assessed the neuroimaging features of mTBI, and they were blinded to the primary end-point.

### Statistical analysis

Uni- and multi-variate logistic regression analyses were adopted to assess the independent correlation between D-dimer at admission and early endotracheal intubation in mTBI patients. The likelihood of early endotracheal intubation is expressed as an odds ratio (OR) with a standard error and 95% confidence interval (95% CI). Subsequently, subgroup analyses were stratified based on pertinent effect covariates, and a forest plot was used to visualize the study effects.

Descriptive scrutiny was performed on all participants. Continuous variables were presented as mean and standard deviation or median and interquartile range based on appropriateness. Categorical variables are described as proportions (%). To compare the demographic characteristics of participants in the presence or absence of tracheal intubation, either the Mann-Whitney U test or the chi-square test was utilized.

Diverse models were evaluated by gradually adjusting for different risk factors. The crude model remained unadjusted, whereas the adjusted model was additionally adjusted for age, sex, GCS at admission, Fisher scales, and ISS. D-dimer was dichotomized into low- and high-level groups based on its median value of 8.7 mg/L.

All statistical analyses were conducted using R (The R Foundation for Statistical Computing, Vienna, Austria) and Free Statistics software (version 1.5; Beijing, China). A significance level of 0.05 was considered statistically significant.

### Sensitivity analyses

To enhance the robustness of our findings, we conducted a propensity score matching (PSM) analysis. A 1:1 nearest neighbor matching method was implemented, and a caliper width of 0.2 was used. A multi-variable logistic regression model was applied to compare patients with low-level (<8.7) and high-level (≥8.7) D-dimer groups based on its median. Variables selected for generating the propensity score included age, GCS, Marshall scores, and ISS. PSM balance was evaluated using a standardized mean difference. A threshold of <0.1 was deemed acceptable.

## Results

### Patient population

**FIG. 1.**
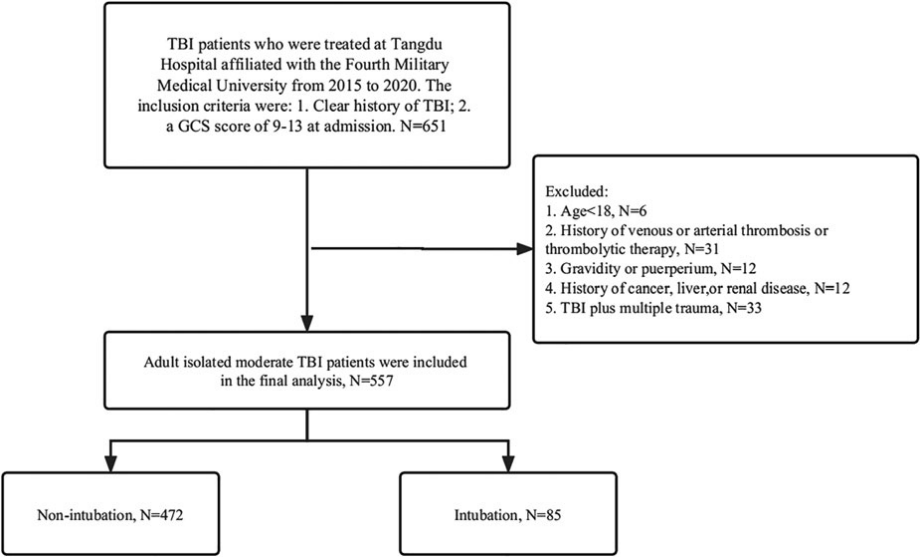
Flowchart that illustrates the selection process. Records of moderate traumatic brain injury patients enrolled in Tangdu Hospital from 2015 to 2020 with criteria of inclusion and exclusion. GCS, Glasgow Coma Scale; TBI, traumatic brain injury.

A total of 651 participants were initially screened. After the application of inclusion and exclusion criteria, a total of 557 mTBIs were included for analysis. Figure 1 shows the reasons for excluding some patients from the analysis.

### Baseline characteristics

Demography, clinical characteristics, and primary outcome of all participants are presented in Table 1. These participants were divided into four level groups based on the median and interquartile range of the D-dimer levels: level 1, level 2, level 3, and level 4, and the intubation rate between the four levels was statistically significant (*p* < 0.001). Among demographic and clinical characteristics, age, the percentage of low GCS scores (*p* = 0.012) and of high Marshall scores (*p* = 0.019), ISS (*p* < 0.001), white blood cell (WBC) count (*p* < 0.001), platelet count (*p* = 0.025), fibrin degradation products (FDPs; *p* < 0.001), activated partial prothrombin time (APTT; *p* = 0.010), aspartate aminotransferase (AST; *p* < 0.001), and D-dimer levels (*p* < 0.001) were significantly increased from the level 1 group to the level 4 group. In contrast, red blood cell count (*p* = 0.093), hemoglobin level (*p* = 0.272), alanine aminotransferase (ALT; *p* = 0.291), creatine (*p* = 0.296), prothrombin time (PT; *p* = 0.817), the percentage of male (*p* = 0.961), smoking (*p* = 0.478) or alcohol history (*p* = 0.934), MLS (*p* = 0.206), and comorbidities, including COPD (*p* = 0.542), HTN (*p* = 0.082), CAD (*p* = 0.916), and DM (*p* = 0.076), remained stable across all groups (*p* > 0.05; Table 1).

**Table 1.** Demography and Clinical Characteristics Based on Four Levels of D-Dimer in Moderated Traumatic Brain Injury

Variables	Total	Level 1	Level 2	Level 3	Level 4	*p *value
*N*	557	140	142	136	139	
Age	53 (41, 63)	52 (40, 61)	51 (40, 62)	54 (42, 62)	58 (47, 64)	0.010^*^
Male	399 (71.6)	101 (72.1)	103 (72.5)	95 (69.9)	100 (71.9)	0.961
COPD	49 (8.8)	10 (7.1)	10 (7)	15 (11)	14 (10.1)	0.542
HTN	116 (20.8)	23 (16.4)	40 (28.2)	26 (19.1)	27 (19.4)	0.082
CAD	26 (4.7)	5 (3.6)	7 (4.9)	7 (5.1)	7 (5)	0.916
DM	43 (7.7)	7 (5)	18 (12.7)	9 (6.6)	9 (6.5)	0.076
GCS						0.012^*^
9	72 (12.9)	13 (9.3)	13 (9.2)	27 (19.9)	19 (13.7)	
10	130 (23.3)	23 (16.4)	33 (23.2)	35 (25.7)	39 (28.1)	
11	96 (17.2)	34 (24.3)	24 (16.9)	15 (11)	23 (16.5)	
12	122 (21.9)	27 (19.3)	33 (23.2)	33 (24.3)	29 (20.9)	
13	137 (24.6)	43 (30.7)	39 (27.5)	26 (19.1)	29 (20.9)	
Smoking	79 (14.2)	18 (12.9)	24 (16.9)	15 (11)	22 (15.8)	0.478
Alcohol	45 (8.1)	11 (7.9)	11 (7.7)	10 (7.4)	13 (9.4)	0.934
Marshall scores						0.019^*^
1	101 (18.1)	39 (27.9)	26 (18.3)	18 (13.2)	18 (12.9)	
2	353 (63.4)	82 (58.6)	86 (60.6)	97 (71.3)	88 (63.3)	
3	29 (5.2)	6 (4.3)	10 (7)	3 (2.2)	10 (7.2)	
4	5 (0.9)	1 (0.7)	0 (0)	2 (1.5)	2 (1.4)	
5	69 (12.4)	12 (8.6)	20 (14.1)	16 (11.8)	21 (15.1)	
MLS	96 (17.2)	16 (11.4)	27 (19)	25 (18.4)	28 (20.1)	0.206
ISS	11 (11, 14)	11 (9, 11)	11 (11, 14)	11 (11, 14)	14 (11, 19)	<0.001^*^
RBC	4.3 ± 0.7	4.2 ± 0.7	4.4 ± 0.7	4.3 ± 0.7	4.2 ± 0.6	0.093
WBC	13.4 (9.9, 17.3)	11.2 (7.9, 13.6)	13.5 (9.9, 17.4)	14.5 (11.0, 17.5)	15.2 (12.9, 19.7)	<0.001^*^
Hemoglobin	134.1 ± 20.6	134.7 ± 21.4	136.5 ± 20.8	133.6 ± 20.7	131.7 ± 19.5	0.272
Platelet	186.0 ± 64.3	195.8 ± 68.5	187.5 ± 60.4	172.6 ± 64.0	187.5 ± 62.6	0.025^*^
PT	11.6 ± 1.6	11.6 ± 1.7	11.6 ± 1.5			0.817
FDP	18.8 (7.4, 40.6)	3.6 (2.2, 5.8)	12.9 (9.3, 15.8)	27.5 (22.2, 34.4)	70.2 (51.2, 100.0)	<0.001^*^
APTT	24.5 ± 5.4	25.8 ± 4.8	24.4 ± 4.8	23.7 ± 5.9	24.2 ± 5.7	0.010^*^
D-dimer	8.7 (3.0, 17.9)	1.5 (0.9, 2.1)	5.9 (4.2, 7.4)	12.5 (10.2, 15.0)	32.1 (21.8, 45.0)	<0.001^*^
AST	39.0 (31.0, 54.0)	33.5 (28.0, 47.8)	37.0 (30.0, 54.0)	39.0 (32.0, 49.0)	45.0 (36.0, 66.0)	<0.001^*^
ALT	31.0 (24.0, 43.0)	31.0 (25.0, 44.2)	31.5 (24.0, 44.2)	31.0 (22.2, 39.0)	32.0 (26.0, 44.0)	0.291
Creatine	55.9 (47.9, 68.4)	55.1 (48.3, 65.3)	57.6 (49.1, 69.6)	53.8 (46.5, 66.5)	57.7 (48.5, 69.3)	0.296
Intubation	85 (15.3)	9 (6.4)	18 (12.7)	21 (15.4)	37 (26.6)	<0.001^*^

Data are expressed as a number (percentage) or median (quartile 1, quartile 3). D-dimer was divided into four levels based on the median and interquartile range of all the patients' D-dimer data: level 1 (<3.0 mg/L), level 2 (3.0–8.7 mg/L), level 3 (8.7–17.9 mg/L), and level 4 (≥17.9 mg/L).

^*^ The *p* value is under 0.05, which is considered statistically significant.

GCS, Glasgow Coma Scale; CAD, coronary heart disease; HTN, hypertension; DM, diabetes mellitus; COPD, chronic obstructive pulmonary disease; RBC, red blood cell; WBC, white blood cell; MLS, midline shift; DAI, diffuse axonal injury; ISS, Injury Severity Score; FDP, fibrin degradation products; PT, prothrombin time; APTT, activated partial prothrombin time; AST, aspartate aminotransferase; ALT, alanine aminotransferase.

### Association between high levels of D-dimer and the risks for intubation

The association between elevated D-dimer levels and the risk for intubation was examined. In the extended multi-variable logistic regression models (Table 2), significant ORs were consistently observed for high-level D-dimer (levels 3–4) across all five models (*p* < 0.05 for all). After adjusting for age, sex, GCS, Marshall score, ISS, WBC, FDP, and platelet counts, a high risk of intubation was demonstrated in patients whose D-dimer levels were ≥17.9 mg/L (level 4; OR = 3.10; 95% CI, 1.16–8.25; *p* = 0.024; model 4, Table 2).

**Table 2.** ORs and 95% CIs for Intubation Associated With D-Dimer in Moderate Traumatic Brain Injury Patients

Regression model	OR	95% CI	*p *value
Crude model			
Level 1	1 (Ref^1^)	1 (Ref)	
Level 2	2.11	0.91–4.88	0.080
Level 3	2.66	1.17–6.04	0.019^*^
Level 4	5.28	2.44–11.44	<0.001^*^
Model 1			
Level 1	1 (Ref)	1 (Ref)	
Level 2	2.11	0.91–4.88	0.080
Level 3	2.66	1.17–6.04	0.019^*^
Level 4	5.28	2.44–11.44	<0.001^*^
Model 2			
Level 1	1 (Ref)	1 (Ref)	
Level 2	2.18	0.91–5.21	0.080
Level 3	2.05	0.87–4.86	0.103
Level 4	5.19	2.30–11.70	<0.001^*^
Model 3			
Level 1	1 (Ref)	1 (Ref)	
Level 2	2.06	0.85–5.01	0.110
Level 3	1.98	0.82–4.78	0.128
Level 4	4.37	1.87–10.21	0.001^*^
Model 4			
Level 1	1 (Ref)	1 (Ref)	
Level 2	2.07	0.84–5.05	0.112
Level 3	1.84	0.75–4.53	0.183
Level 4	3.10	1.16–8.25	0.024^*^

Model 1 adjusted for age and sex. Model 2 adjusted for model 1 plus GCS. Model 3 adjusted for model 2 plus Marshall score and ISS. Model 4 adjusted for model 3 plus WBC, FDP, and platelet counts. Ref means reference.

^*^ The *p* value is under 0.05, which is considered statistically significant.

OR, odds ratio; 95% CI, 95% confidence interval; GCS, Glasgow Coma Scale; ISS, Injury Severity Score; WBC, white blood cell; FDP, fibrin degradation product.

### Sensitive analysis

**FIG. 2.**
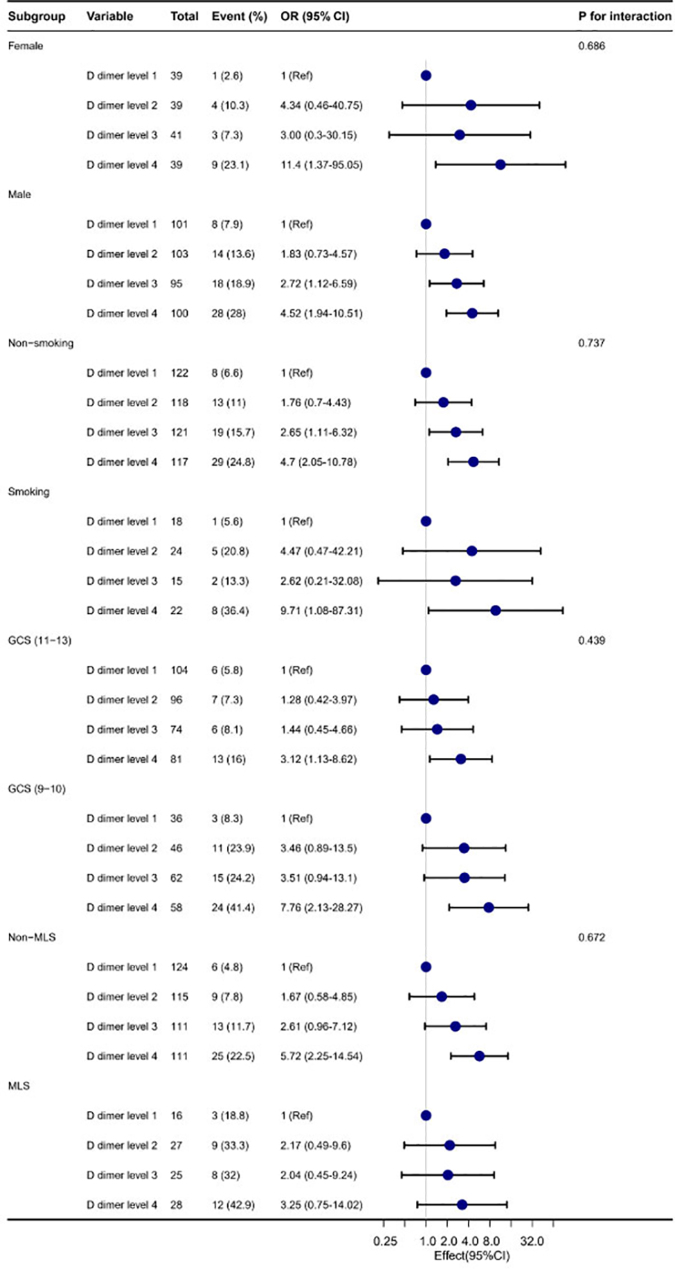
Subgroup analysis of the association between four distinct tiers of D-dimer levels and the occurrence of intubation in moderate traumatic brain injury, using level 1 as the baseline reference level. Each stratification was adjusted for age, sex, GCS, Marshall scores, and ISS. The interaction was not significant in the subgroup. Blue dots: odds ratio; blue lines: 95% confidence interval. 95% CI, 95% confidence interval; GCS, Glasgow Coma Scale; ISS, Injury Severity Score; MLS, midline shift; OR, odds ratio.

Subgroup assessments were conducted, considering confounding factors such as sex, low and high GCS scores, smoking history, and MLS. No significant interaction was detected among the subgroups (*p* value for interaction, >0.05 for all; Fig. 2).

After PSM, 210 pairs in each group were well matched (Supplementary Table S1; Supplementary Fig. S1). Among these 210 propensity-matched pairs, the rate of intubation was significantly higher in the high-level D-dimer group (levels 3–4; 19 [9%] vs. 38 [18%]; *p* = 0.007; Supplementary Table S1). Additionally, the OR remained similar after PSM (OR = 2.22; 95% CI, 1.23–4.00; *p* = 0.008; Table 3). Inverse probability weighting also showed a significantly higher OR in the high-level D-dimer group (OR = 2.05; 95% CI, 1.25–3.36; *p* = 0.004; Table 3).

**Table 3.** Associations of High-Level D Dimer With the Early Intubation in the Propensity Score Analyses

Analysis	OR	95% CI	*p *values
Multi-variable analysis			
With inverse probability weighting^a^	2.05	1.25 ∼ 3.36	0.004^*^
With matching^b^	2.22	1.23 ∼ 4.00	0.008^*^
Adjusted for propensity score^c^	1.84	1.09 ∼ 3.10	0.022^*^

D-dimer was divided into low-level (<8.7 mg/L) and high-level (≥8.7 mg/L) groups for propensity score analyses, based on the median value of all patients' D-dimer.

^*^ The *p* value is under 0.05, which is considered statistically significant.

^a^ Primary analysis with a multi-variable logistic regression model with the same strata and covariates, with inverse probability weighting according to the propensity score.

^b^ Multi-variable logistic regression model with the same strata and covariates, with matching according to the propensity score. The analysis included 420 patients (210 low-level D-dimer and 210 high-level D-dimer).

^c^ Multi-variable logistic regression model with the same strata and covariates, with additional adjustment for the propensity score.

OR, odds range; 95% CI, 95% confidence interval.

## Discussion

This study investigated the connection between D-dimer levels and tracheal intubation in a large cohort of mTBI patients from a single institution. The findings showed that high-level D-dimer (>17.9 mg/L) was independently associated with a greater risk of intubation. The association was consistent in several subgroup analyses and PSM analysis. These results suggest that neurosurgeons or neurointensivists should closely monitor mTBI patients with high-level D-dimer at admission and prioritize them for intensive airway management. Additionally, they should evaluate the need for intubation in a timely manner to prevent hypoxia.

The association between high-level D-dimer and tracheal intubation may be related to neurological deterioration or hypoxemia post-TBI. First, the presence and progression of hemorrhagic injury (PHI) is one of the most common causes of neurological deterioration in patients with mTBI.^1,6–8^ Elevated D-dimer is significantly correlated with PHI in the TBI population^9,10,15–17^; thus, elevated D-dimer is considered a blood biomarker for neurological deterioration.^9,10,15,16^ Because tracheal intubation is a crucial step in managing airway protection after neurological deterioration, the association between elevated D-dimer and intubation can be understood in mTBI patients.

Second, elevated D-dimer levels have been proposed as a potential biomarker for brain injury in isolated TBI patients.^1-4,6,14^ Eiichi and colleagues showed a significant correlation between D-dimer values at admission and soluble tissue factor (sTF) levels in the blood (*R* = 0.803, *p* = 0.009). sTF is released from damaged brain tissue and is a biomarker for brain injury.^6^ The more severe the brain injury, the worse the airway contouring ability, and thus the greater the likelihood of tracheal intubation.^18^ Therefore, elevated D-dimer levels may serve as a potential risk factor for intubation in mTBI patients. However, it is important to note that elevated D-dimer levels can also occur in patients with multiple injuries or thrombosis,^2^ which may limit its use as a specific biomarker for acute isolated TBI patients.

Third, elevated D-dimer levels (>1.5 mg/L) upon admission were also found to be associated with an increased risk of acute respiratory failure (ARF) and need for invasive mechanical ventilation in a nation-wide study of COVID-19 patients in China.^4^ The mechanism between elevated D-dimer and ARF was probably associated with a micro pulmonary embolism (PE), particularly in those with a critical case of COVID-19.^3,19^ There are several studies suggesting that TBI patients may be at an increased risk of developing a PE compared to the general trauma population,^20,21^ because TBI can lead to either a prothrombotic or a prohemorrhagic condition.^22^ Both circumstances can induce embolus, possibly causing pulmonary microvascular occlusion.^23^ There is limited research available on the incidence of PE development in persons with TBI.^20,24,25^ Different population studies reported that the incidence of PE in TBI was from 0.3% to 17%.^20,21,24^ Because of the challenges in making a definitive diagnosis, we could ot ascertain whether PE was the cause of hypoxemia and intubation in our study. Therefore, in the event of an elevated D-dimer, it is imperative to have a high index of suspicion of PE,^26^ priority to receive the diagnostic protocol of PE, and assess the indication of tracheal intubation in time to prevent hypoxia.

Drawing upon the preceding discourse, it becomes apparent that escalated D-dimer levels are interconnected with the advancement of hemorrhagic injury, exacerbation of cerebral impairment, and onset of ARF or PE. The management of these complexities necessitates tracheal intubation, a therapeutic intervention that substantially augments patient outcomes. Consequently, it stands to reason that the adoption of D-dimer-guided intubation holds promise for patients afflicted with mTBI. Nonetheless, the substantiation of this deduction mandates verification by prospective, well-controlled clinical trials.

This study has several strengths. It offers epidemiological evidence of a significant association between elevated D-dimer levels and the likelihood of tracheal intubation, based on a large study population. Further, other than the common confounders (age, sex, smoking, and comorbidities), we also examined the effects of neurological variables on intubation, such as GCS, Marshall scores at admission, MLS, and ISS. Last, we conducted sensitivity analyses to ensure the robustness of our findings.

There are also several noteworthy limitations. In the first place, its retrospective design is susceptible to confounding variables. Residual confounding variables, such as delirium, agitation, and sedative use, potentially exist, although we took steps to adjust for potential confounders and reduced the impact of factors that could cause an outcome bias. Second, given that the study population only contained patients with acute isolated mTBI (GCS 9–13), it may not be extrapolated to all the TBI population. Third, the limitations of a retrospective study prevented us from obtaining imaging evidence of a micro PE, which would be useful to determine its role in the association between D-dimer and ARF.

## Conclusion

Within this extensive cohort of mTBI patients, elevated D-dimer levels were observed to exhibit an autonomous correlation with an escalated susceptibility to intubation. This correlation demonstrated resilience across diverse subgroup analyses and subsequent PSM evaluations. Nevertheless, the advantageous outcomes ensuing from D-dimer-driven intubation remain in a state of ambiguity. Consequently, it becomes imperative to undertake additional prospective clinical investigations to ascertain the utility of D-dimer-guided intubation for persons with mTBI.

## Supplementary Material

Supplemental data

Supplemental data

## Transparency, Rigor, and Reproducibility Summary

This study was not formally registered because it was a retrospective study. The analysis plan was not formally registered. A sample size of 557 subjects in the group was planned based on an expected effect size of 85 for the primary outcome measure, calculated to yield >80% power to detect intubation using multiple logistic regression with a *p* value <0.05. A total of 651 of participants were screened. Data collection was performed by investigators blinded to relevant participant characteristics such as pre-defined group membership. Data were acquired between January 2015 and December 2020. Data were collected using the EHR system of the healthcare facility and were analyzed using R (The R Foundation for Statistical Computing, Vienna, Austria) and Free Statistics software (version 1.5; Beijing, China). All data sets were analyzed at the same time. The time required for the data acquisition was from July 2022 to October 2022. Data collection and analysis were performed on different computers. All equipment and software used to perform acquisition and analysis are widely available from laboratories. The key inclusion criteria are established standards in the field.

The statistical tests used were based on the assumptions that D-dimers are an independent risk factor for early intubation. This study was not required for multiple comparisons. No replication or external validation studies have been performed or are ongoing at this time to our knowledge. The data sets generated during and/or analyzed during the current study are available from the corresponding author on reasonable request. Analytical code used to conduct the analyses presented in this study are not available for public use because of intellectual property. The authors agree or have agreed to publish the manuscript using the Mary Ann Liebert Inc. “Open Access” option under the appropriate license.

## Abbreviations Used

ALT: alanine aminotransferase
APTT: activated partial prothrombin time
ARF: acute respiratory failure
AST: aspartate aminotransferase
CAD: coronary heart disease
95% CI: 95% confidence interval
COPD: chronic obstructive pulmonary disease
DM: diabetes mellitus
HER: electronic health record
FDP: fibrin degradation product
GCS: Glasgow Coma Scale
HTN: hypertension
ISS: Injury Severity Score
MLS: midline shift
mTBI: moderate TBI
OR: odds ratio
PE: pulmonary embolism
PHI: progression of hemorrhagic injury
PSM: propensity score matching
PT: prothrombin time
sTF: soluble tissue factor
TBI: traumatic brain injury
WBC: white blood cell
