# Supplementary material for: Association Between High-Level D-Dimer at Admission and Early Intubation in Patients With Moderate Traumatic Brain Injury
Source: Neurotrauma Rep. 2023 Oct 25;4(1):715–23. doi: 10.1089/neur.2023.0068 (PMC10615076; doi:10.1089/neur.2023.0068)
Supplement: Supplemental data [file Suppl_TableS1.docx]

**Supplement materials**

Table S1: Study participants’ clinical characteristics in full cohort and after propensity score matching.

| **Unmatched** | | | | | **Matched (1:1)** | | | |
| --- | --- | --- | --- | --- | --- | --- | --- | --- |
| **Item** | **Low-level D-dimer** | **High-level D-dimer** | ***p*** | **Low-level D-dimer** | | **High-level D-dimer** | ***p*** |  |
| n | 282 | 275 |  | 210 | | 210 |  |  |
| Age | 51 (40, 61) | 56 (44, 64) | 0.002 | 53 (42, 63) | | 53 (42, 62) | 0.627 |  |
| Male/% | 204 (72.3) | 195 (70.9) | 0.708 | 141 (70.1) | | 144 (71.6) | 0.742 |  |
| COPD/% | 20 (7.1) | 29 (10.5) | 0.15 | 14 (7) | | 20 (10) | 0.282 |  |
| HTN/% | 63 (22.3) | 53 (19.3) | 0.373 | 58 (28.9) | | 32 (15.9) | 0.002^*^ |  |
| CAD/% | 12 (4.3) | 14 (5.1) | 0.64 | 12 (6) | | 4 (2) | 0.041^*^ |  |
| DM/% | 25 (8.9) | 18 (6.5) | 0.305 | 22 (10.9) | | 11 (5.5) | 0.046^*^ |  |
| GCS/% |  |  | 0.002 |  | |  | 0.992 |  |
| 9 | 26 (9.2) | 46 (16.7) |  | 25 (12.4) | | 24 (11.9) |  |  |
| 10 | 56 (19.9) | 74 (26.9) |  | 49 (24.4) | | 48 (23.9) |  |  |
| 11 | 58 (20.6) | 38 (13.8) |  | 36 (17.9) | | 36 (17.9) |  |  |
| 12 | 60 (21.3) | 62 (22.5) |  | 50 (24.9) | | 48 (23.9) |  |  |
| 13 | 82 (29.1) | 55 (20) |  | 41 (20.4) | | 45 (22.4) |  |  |
| Smoking/% | 42 (14.9) | 37 (13.5) | 0.626 | 35 (17.4) | | 29 (14.4) | 0.413 |  |
| Acohol/% | 22 (7.8) | 23 (8.4) | 0.808 | 15 (7.5) | | 20 (10) | 0.376 |  |
| Marshall scores/% |  |  | 0.017 |  | |  | 0.599 |  |
| 1 | 65 (23) | 36 (13.1) |  | 27 (13.4) | | 29 (14.4) |  |  |
| 2 | 168 (59.6) | 185 (67.3) |  | 133 (66.2) | | 137 (68.2) |  |  |
| 3 | 16 (5.7) | 13 (4.7) |  | 13 (6.5) | | 7 (3.5) |  |  |
| 4 | 1 (0.4) | 4 (1.5) |  | 1 (0.5) | | 0 (0) |  |  |
| 5 | 32 (11.3) | 37 (13.5) |  | 27 (13.4) | | 28 (13.9) |  |  |
| MLS/% | 43 (15.2) | 53 (19.3) | 0.209 | 36 (17.9) | | 31 (15.4) | 0.503 |  |
| ISS | 11.2 ± 4.1 | 14.0 ± 5.5 | <0.001 | 12.3 ± 4.1 | | 12.3 ± 4.2 | 0.877 |  |
| HBG | 135.6 ± 21.1 | 132.7 ± 20.1 | 0.095 | 133.9 ± 21.3 | | 134.0 ± 20.6 | 0.972 |  |
| RBC | 4.3 ± 0.7 | 4.2 ± 0.6 | 0.265 | 4.3 ± 0.7 | | 4.3 ± 0.6 | 0.779 |  |
| WBC | 12.1 (8.6, 15.9) | 14.8 (12.0, 18.3) | <0.001 | 11.8 (8.5, 15.3) | | 14.9 (12.1, 18.2) | <0.001^*^ |  |
| Platelet | 191.6 ± 64.6 | 180.1 ± 63.6 | 0.035 | 190.9 ± 64.6 | | 182.8 ± 58.2 | 0.188 |  |
| FDP | 7.8 (3.6, 13.9) | 40.7 (27.8, 73.7) | <0.001 | 8.1 (4.4, 14.3) | | 40.7 (27.3, 66.7) | <0.001^*^ |  |
| D-dimer | 3.1 (1.6, 6.0) | 18.2 (12.5, 32.4) | <0.001 | 3.3 (1.6, 6.0) | | 17.9 (12.2, 29.0) | <0.001^*^ |  |
| PT | 11.4 ± 1.5 | 11.8 ± 1.7 | 0.01 | 11.5 ± 1.7 | | 11.8 ± 1.6 | 0.189 |  |
| APTT | 25.1 ± 4.8 | 24.0 ± 5.8 | 0.016 | 25.1 ± 4.9 | | 23.9 ± 5.9 | 0.021^*^ |  |
| Intubation | 27 (9.6) | 58 (21.1) | <0.001 | 19 (9) | | 38 (18) | 0.007^*^ |  |

Date is expressed as a number (percentage) or median (quartile1, quartile3). GCS: Glasgow Coma Scale. CAD: coronary heart disease. HTN: hypertension. DM: diabetes mellitus. COPD: chronic obstructive pulmonary disease. MLS: midline shift. DAI: diffused axion injury. RBC: red blood cell. WBC: white blood cell. HGB: Hemoglobin. ISS: injury severity score. PT: prothrombin time. APTT: activated partial prothrombin time. FDP: fibrin degradation products.

^*^ The *p* value is under 0.05, which is considering statistically significant.
