# Supplementary material for: Association Between High-Level D-Dimer at Admission and Early Intubation in Patients With Moderate Traumatic Brain Injury
Source: Neurotrauma Rep. 2023 Oct 25;4(1):715–23. doi: 10.1089/neur.2023.0068 (PMC10615076; doi:10.1089/neur.2023.0068)
Supplement: Supplemental data [file Suppl_FigS1.docx]

**Figure S1. The standardized mean difference values between before and after propensity score matching**


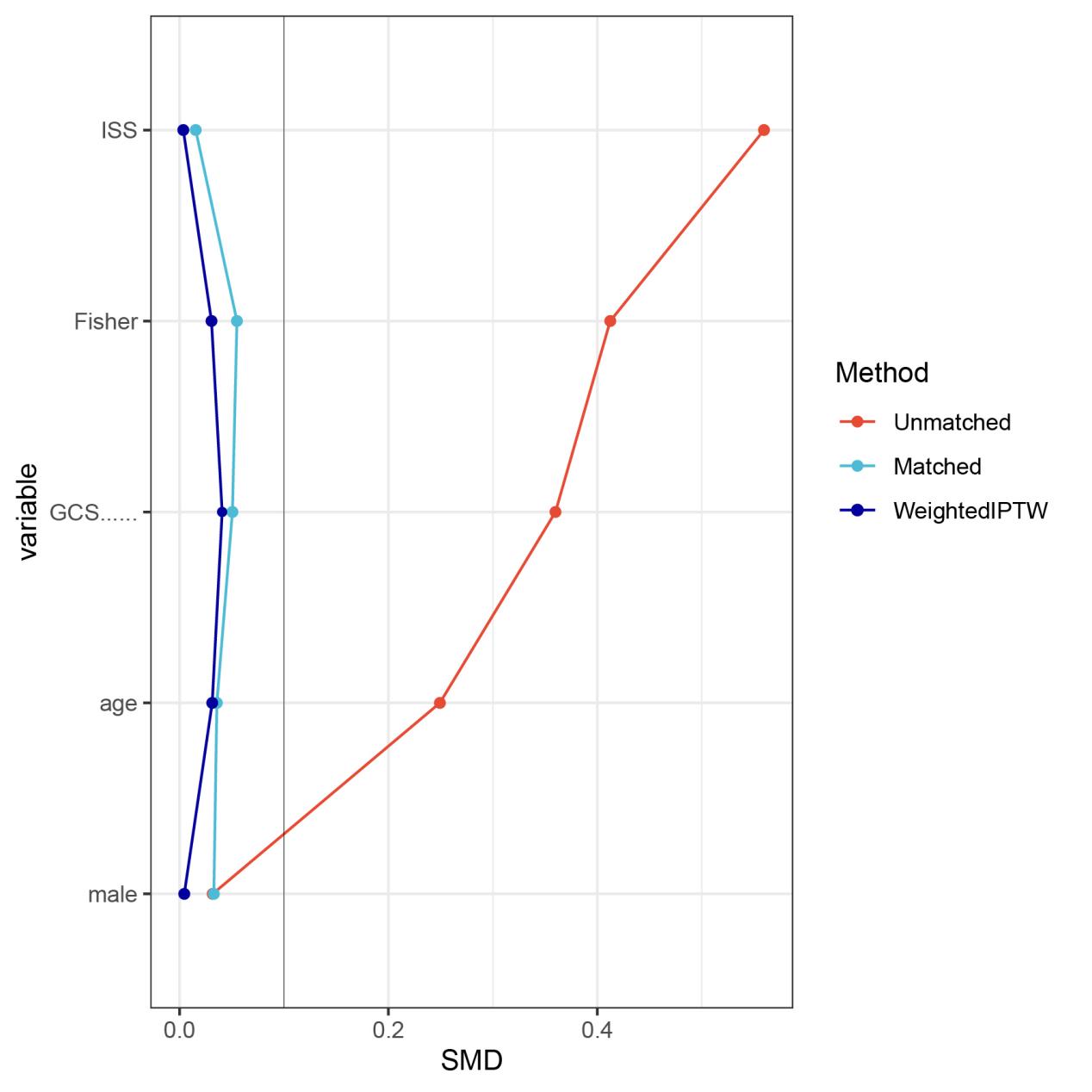


The PSM degree was estimated by a standardized mean difference (SMD). A threshold < 0.1 was considered acceptable. IPTW: Inverse probability of treatment weighting.
